# Repurposing Antiviral Protease Inhibitors Using Extracellular Vesicles for Potential Therapy of COVID-19

**DOI:** 10.3390/v12050486

**Published:** 2020-04-26

**Authors:** Santosh Kumar, Kaining Zhi, Ahona Mukherji, Kelli Gerth

**Affiliations:** 1 Department of Pharmaceutical Sciences, University of Tennessee Health Science Center, 881 Madison Ave, Memphis, TN 38163, USA; imahona@gmail.com (A.M.); kgerth1@uthsc.edu (K.G.); 2 Plough Center for Sterile Drug Delivery Solutions, University of Tennessee Health Science Center, 208 South Dudley Street, Memphis, TN 38163, USA; kzhi@uthsc.edu

**Keywords:** COVID-19, coronaviruses, antiviral drugs, HIV, protease inhibitors, extracellular vesicles

## Abstract

In January 2020, Chinese health agencies reported an outbreak of a novel coronavirus-2 (CoV-2) which can lead to severe acute respiratory syndrome (SARS). The virus, which belongs to the coronavirus family (SARS-CoV-2), was named coronavirus disease 2019 (COVID-19) and declared a pandemic by the World Health Organization (WHO). Full-length genome sequences of SARS-CoV-2 showed 79.6% sequence identity to SARS-CoV, with 96% identity to a bat coronavirus at the whole-genome level. COVID-19 has caused over 133,000 deaths and there are over 2 million total confirmed cases as of 15 April 2020. Current treatment plans are still under investigation due to a lack of understanding of COVID-19. One potential mechanism to slow disease progression is the use of antiviral drugs to either block the entry of the virus or interfere with viral replication and maturation. Currently, antiviral drugs, including chloroquine/hydroxychloroquine, remdesivir, and lopinavir/ritonavir, have shown effective inhibition of SARS-CoV-2 in vitro. Due to the high dose needed and narrow therapeutic window, many patients are experiencing severe side effects with the above drugs. Hence, repurposing these drugs with a proper formulation is needed to improve the safety and efficacy for COVID-19 treatment. Extracellular vesicles (EVs) are a family of natural carriers in the human body. They play a critical role in cell-to-cell communications. EVs can be used as unique drug carriers to deliver protease inhibitors to treat COVID-19. EVs may provide targeted delivery of protease inhibitors, with fewer systemic side effects. More importantly, EVs are eligible for major aseptic processing and can be upscaled for mass production. Currently, the FDA is facilitating applications to treat COVID-19, which provides a very good chance to use EVs to contribute in this combat.

## 1. Introduction

Coronavirus disease 2019 (COVID-19) is a current, emerging infectious disease; it has been declared a pandemic by the World Health Organization (WHO). COVID-19 is caused by severe acute respiratory syndrome coronavirus 2 (SARS-CoV-2) [1]. Dr. Zhengli Shi, the most famous scientist in the field of SARS, has proposed that the origin of SARS-CoV-2 could be from bats in Yunan Province, which is 2000 km away from Wuhan, in Hubei province [2]. Based on the history of SARS, Middle East Respiratory Syndrome (MERS), and Swine Acute Diarrhea Syndrome (SADS), two of which originated from China through bats, researchers in China in early 2019 speculated that SARS- or MERS-like coronaviruses are likely to originate from bats in China [3,4]. Although the immediate origin and transfer to humans is debatable, rapid human-to-human transfer has been widely confirmed. COVID-19 causes symptomatic severe acute respiratory disease in approximately 15% of infected individuals and fatality in approximately 4%, though these rates vary from country to country [1].

The previous two zoonotic coronaviruses that caused a worldwide pandemic are MERS and SADS, which appeared in 2012 and 2017, respectively [5]. Compared to these coronaviruses and other related viruses like Ebola (2003) and H1N1 (2009), SARS-CoV-2 has emerged as the most resilient, with a perfect combination of ease of transmission, late incubation period, symptomatic nature, and morbidity and mortality [6]. Statistically, a very small percentage of viruses, even among coronaviruses, will have the right combination of infection rate, incubation period, and morbidity and mortality [7]. SARS-CoV-2 is transmitted human-to-human through air droplets that result from sneezing, coughing, or even breathing and speaking. This has led to the transmission of this virus among large populations worldwide within a few months. Its long incubation time (5–10 days) makes it difficult to detect early symptoms; thus, asymptomatic persons can inadvertently spread the virus to others. The COVID-19 mortality rate appears to be lower than with other recent viral outbreaks. However, it is essential to note that its mortality rate is difficult to measure accurately, as the data is still being collected. Importantly, it attacks vulnerable populations, such as immunocompromised and elderly individuals, as well as those with underlying conditions, such as heart and lung conditions, diabetes, and kidney disease [1]. COVID-19 is especially deadly among these populations [8].

In many countries, anti-HIV drugs (lopinavir/ritonavir and saquinavir), antimalaria drugs (chloroquine and hydroxychloroquine) and other drugs have been tested in clinics. Some of these drugs have shown potential in reducing the symptoms or treating COVID-19 [9,10,11]. Novel drugs and vaccines are also being developed by many institutions, as well as by biotech and pharmaceutical companies across the world [12,13]. However, it is likely to be at least one year before drugs and/or vaccines become available for administration to COVID-19 patients. Therefore, until we find drugs and/or vaccines for COVID-19, repurposing existing drugs is likely to play a significant role in reducing symptoms or treating the disease in patients. It is, however, important to note that social distancing and taking extra precautions are the principle ways one can mitigate the worldwide spread and morbidity and mortality caused by SARS-CoV-2 infection [14]. In this review, we will first briefly describe the structural and genetic features of SARS-CoV-2, current and predicted epidemiology of COVID-19 worldwide, current treatment options, potential targets in the SARS-CoV-2 life cycle for drug development and/or repurposing of existing drugs, manufacturing feasibility, and regulatory affairs. The main theme of this review, however, is the repurposing of protease inhibitor (PI) drugs, which are currently used to treat Human Immunodeficiency Virus (HIV), Hepatitis C Virus (HCV), and other RNA viruses, in the treatment of COVID-19.

## 2. Epidemiological Data Around the World

Although severe cases of COVID-19 remain the most contagious, both asymptomatic and recovered patients impede efforts to control the spread of the virus. Unique among infectious diseases, patients who have recovered from SARS-CoV-2 infection continue to exhibit positive real-time polymerase chain reaction (RT-PCR) tests [15]. Thus, the Center of Disease Control (CDC) guidelines suggest “social distancing” of at least 6 ft as the primary means of controlling viral spread [16]. A significant loss of human life has already occurred around the world, and new modeling predicts that further, substantial loss is on the horizon.

Since the initial outbreak in Wuhan in December of 2019, over 83,000 cases of COVID-19 were confirmed across mainland China as of 15 April 2020 [15]. The World Health Organization (WHO) declared COVID-19 a “pandemic” on 11 March 2020 [17]. While China has seen rates of infection peak and subside dramatically, confirmed cases are now experiencing exponential growth in Europe and the United States, with an increasing number of cases now observed on the Indian subcontinent and in South America [18]. As of 15 April 2020, The Johns Hopkins Center for Systems Science and Engineering (CSSE) has tracked over 2 million cases worldwide, including 345,000 cases in the United States alone. Additionally, over 133,000 deaths and over 350,000 recoveries have been observed worldwide. The United States now leads the world in the total number of confirmed cases [19]. Assuming that full social distancing is enforced in the United States through the end of May 2020, modeling from the University of Washington’s Institute for Health Metrics and Evaluation (IHME) predicts that daily deaths will peak on 16 April 2020 at 2644 deaths per day. The curve will decay through May and June, and will not flatten until July. By 4 August IHME projects a total of 93,531 deaths from COVID-19 in the United States alone [20].

It is important to note that the total number of confirmed cases worldwide may not reflect the actual number, due in part to limitations in testing and undetected cases [21,22]. Therefore, a high likelihood that large numbers of cases remain undetected only increases the urgency of facilitating widespread testing and finding sustainable treatment strategies for COVID-19.

## 3. Structural and Genetic Features of COVID-19

Similar to other coronaviruses, SARS-CoV-2 is a spherical particle, approximately 50 nm in diameter. It contains a membrane, envelope protein, nucleocapsid, and most importantly, “spike” protein projecting from its surface, as well as single-stranded RNA (ssRNA) [23]. The SARS-CoV-2 spike protein binds to a receptor on the human cell surface called angiotensin-converting enzyme 2 (ACE2), which is most abundant in the type II alveolar cells of the lungs [24,25]. Latching of the spike protein onto human cells causes them to undergo a structural change that allows the viral membrane to fuse with the host cell membrane. Viral genes then enter into the host cells and translate into polypeptides, which further mature into the individual proteins required to form the viral core and surface protein spike. The virus then matures, produces multiple copies, and escapes from the host cells to infect new cells.

The genome of SARS-CoV-2 is 79.6% identical to other coronaviruses genomes, with as much as 95% identity at a certain part of the genome of other coronaviruses and with bat coronaviruses [2]. SARS-CoV-2 encodes four structural genes (spike, envelope, membrane, and nucleocapsid). The largest gene in SARS-CoV-2 is orflab (open reading frames 1a and 1b), encoding the pplab (peptide pheromone encoding lipoprotein A and B) and 15 nsps (viral nonstructural proteins). This also encodes for the ppla protein (peptide pheromone encoding lipoprotein A), which contains 10 nsps. The absence of 8a protein and fluctuation in the number of amino acids in the 8b and 3c proteins in SARS-CoV-2 differentiates it from other coronaviruses. The major factor which makes SARS-CoV-2 more infective than other coronaviruses is its spike protein, which is highly specific to binding human ACE2 enzyme [26]. A single N56IT mutation significantly enhances the binding affinity of spike protein of SARS-CoV-2with ACE2.

## 4. Potential Target for Drug Development for COVID-19

Based on the known life cycle of SARS-CoV-2, the WHO has proposed several targets for drug development, as well as the repurposing of existing drugs [27]. The first target is attacking the virus with monoclonal antibodies or convalescent plasma (plasma obtained from recovered patients). The second target is inhibition of ACE2 by using novel or existing drugs, which may have cross-reactivity with other ACEs. The third target could be the inhibition of viral endocytosis by using antimalaria drugs, e.g., chloroquine or hydroxychloroquine. The fourth potential and important target could be inhibition of proteolysis of polypeptides, using general or specific proteases. Many anti-HIV, anti-HCV, and other antiviral and antiretroviral drugs have been developed to target proteases [28,29]. Finally, the fifth target could be the inhibition of RNA polymerization using RNA polymerase, developed for other RNA viruses. This could inhibit the formation of multiple copies of RNA, and ultimately, viral replication.

## 5. Potential Treatment of COVID-19

Due to the current absence of effective therapies for COVID-19, several existing drugs that are known to treat other RNA viruses, such as HIV and other coronaviruses, in addition to antimalarial drugs and other drugs used in infectious diseases, have been tried in many countries [30]. Among all available choices, the most promising candidates appear to be plasma and antibody treatments, the anti-Co-V drug remdesivir, the antimalaria drugs chloroquine and hydroxychloroquine, and the anti-HIV drugs lopinavir/ritonavir. The WHO has launched global megatrials of these drugs for coronavirus treatments [31]. The antitumor necrosi factor (TNF) antibody, tocilizumab, has shown benefits in controlling cytokine release syndrome in COVID-19 patients [32]. Healthcare professionals from China and Italy have already started recruiting patients for tocilizumab studies to evaluate its efficacy in COVID-19 patients with severe symptoms. The anti-inflammatory drugs baricitinib and thalidomide were proposed for regulating immunity and inhibiting inflammatory cytokine surge in COVID-19 patients, based on clinical case reports [32,33]. Several clinical studies have registered baricitinib and thalidomide to study anti-inflammatory efficacy in COVID-19 patients. Table 1 is a summary of the most popular drugs that are under investigation to treat COVID-19. 

### 5.1. Convalescent Plasma

In this technique, plasma or purified monoclonal antibodies produced against COVID-19 are obtained from recovered patients and given to new patients as treatment. In a recent study, convalescent plasma treatments were administered to five critically ill COVID-19 patients in Shenzhen, China, from 20 January to 25 March 2020 [34]. In this study, patients received convalescent plasma with a SARS-CoV-2-specific antibody between 10 and 22 days after admission. Among the five patients, four of them showed a decreased score in sequential organ failure assessments and viral loads. Their viral test also became negative within 12 days after the transfusion. These four patients were also removed from mechanical ventilation within 2 weeks of treatment. Finally, three patients were discharged from the hospital in approximately 50 days. Although this trial has a very small sample size, the results of convalescent plasma treatment are still encouraging. This method has been proposed as a treatment option in the US [35].

### 5.2. Tocilizumab

Tocilizumab is a recombinant, humanized antihuman IL-6 receptor monoclonal antibody. It was originally developed to treat rheumatoid arthritis, based on its specific binding to sIL-6R and mIL-6R and inhibition of signal transduction [36]. More importantly, humans have a very high tolerance for Tocilizumab. There were no reported significant abnormalities in clinicopathological studies or histopathological evaluations. Scientists and physicians in China suspect that IL-6 may play a role in the treatment of COVID-19. Their findings concluded that tocilizumab is an effective treatment in patients with severe cases of COVID-19.

### 5.3. Thalidomide

Thalidomide is an immunomodulatory and anti-inflammatory agent. It was designed to boost T cells, treat inflammation, inhibit cell proliferation, and reduce lung injury and pulmonary fibrosis [32]. In COVID-19, its main role is to protect the lungs from damage caused by immunological reactions. However, thalidomide needs to be used with other antiviral agents, since it does not eliminate or suppress viral load. A case report from Wenzhou medical university has proved that thalidomide has adjuvant effects in COVID-19 treatment.

### 5.4. Remdesivir

Remdesivir was originally developed to combat Ebola and related viruses; it works by inhibiting RNA-dependent RNA polymerase [37]. Since the target is similar, both in vitro and in vivo experiments showed that the drug can inhibit the coronaviruses that cause SARS and MERS [38]. The first COVID-19 patient from Washington, U.S. was given remdesivir when his condition worsened, and the case report suggests that his condition improved [39]. Another late-stage patient from California also survived upon receiving Remdesivir. Case reports alone cannot prove that Remdesivir is safe and effective, and therefore, clinical trials are underway. Since it is well-tolerated at relatively high doses, it is possible that Remdesivir is likely to work if given at a high dose and early during the infection, without causing significant toxicity.

### 5.5. Chloroquines

The antimalaria drugs chloroquine and hydroxychloroquine have shown efficacy against COVID-19 in patients and in in vitro experiments. Currently, several countries are recruiting patients to test the safety and efficacy of chloroquine, including Brazil, Spain, Norway, China, and Italy. The first clinical trial for hydroxychloroquine has been completed in Shanghai [40]. The drugs generally work by decreasing the acidity in endosomes, which ingest outside materials, including viruses [41]. Although the entry mechanism of SARS-CoV-2 is through the spike-ACE2 interaction, in vitro studies using cell culture have suggested that chloroquines have inhibition activity against SARS-CoV-2 at relatively high doses, with a potential risk of side effects [42]. Chloroquines also work by inhibiting cytokine storm, which occurs as a result of massive viral replication [43]. The usefulness of chloroquines, especially hydroxychloroquine, may be explained retrospectively by the fact that there is a reduced spreading of SARS-CoV-2 in malaria-rich regions, where hydroxychloroquine has been used on a routine basis. Hydroxychloroquine may work as prophylaxis for COVID-19, depending on the clinical trial results from Shanghai [40]. Opinion among scientists and health care professionals increasingly supports the use of hydroxychloroquine as a therapeutic agent. As a result, the FDA and CDC have allowed hydroxyquinoline as potential therapy for COVID-19 in the clinics upon physician’s prescription with extra precautions.

### 5.6. Protease Inhibitors

Earlier literature suggests that protease inhibitors (PIs), including the alcohol-dependence drug disulfiram and antiviral drugs lopinavir and ritonavir, have been reported to be active against SARS and MERS [44]. Although clinical studies are lacking, disulfiram has been reported to inhibit the papain-like protease of MERS and SARS in cell culture [45]. With regard to the HIV PIs lopinavir and ritonavir, clinical trials in patients infected with 2019-nCoV have been initiated in multiple countries [46]. Lopinavir and ritonavir have initially shown improved clinical outcomes in patients with SARS in a nonrandomized trial. However, it is yet to be determined whether HIV PIs could effectively inhibit the 3-chymotrypsin-like and papain-like proteases of 2019-nCoV [47]. While HIV protease belongs to the aspartic protease family, SARS-CoV-2 proteases are from the cysteine protease family. In a separate study, Martinez showed that LPV/RTV and interferon beta (LPV/RTV-INFb) in combination are effective in patients infected with SARS-CoV [48]. The drugs also showed improved clinical parameters in mice infected with MERS-CoV.

In some countries, including China and India, anti-HIV drugs (LPV/RTV) have been used against COVID-19 [47,49]. Although these drugs showed encouraging results from in vitro studies, data from Wuhan, China using a very small sample size did not show a significant difference [47]. The recipients of these drugs and those receiving standard care did not differ significantly in time to clinical improvement (median: 16 days), duration of intensive care unit stay, days of mechanical ventilation, or days of oxygen support. A similar study by Bhatnagar et al. using LPV/RTV for treating COVID-19 patients in India is underway [49]. If the treatment outcomes amongst initial cases are found to be useful in managing initial COVID-19 patients, a randomized controlled trial would be performed to guide future therapeutic use of this combination.

Another study from Korea by Kim et al. on the use of LPV/RTV in COVID-19 patients suggests that the decrease in viral titer may be a result of an antiviral effect, the natural cause of viral suppression, or both [50,51]. However, the authors are optimistic that LPV/RTV administration reduces viral load. A limitation of the study is that the patients were given LPV/RTV on day 10, when patients have been shown to naturally improve their symptoms, including fever. The effect of LPV/RTV is yet to be seen if the drugs are given early, as soon as individuals are infected by SARS-CoV-2. Regardless of the case report, the authors believe that LPV/RTV are promising anti-HIV drugs for the treatment of COVID-19. However, prior to its recommendation, well-designed studies need to be carried out to gain more evidence.

Although these results are not promising, using an in silico protein-drug modeling approach, Ortega et al. determined that the main SARS-CoV-2 protease is a target for HIV PIs [52,53]. Their results showed a strong interaction between HIV PIs (e.g., LPV and RTV) and the active site of the SARS-CoV-2 protease. They further tested a library of 20 PIs, which revealed potential interactions between the SARS-CoV-2 protease and these PIs. The results suggest development of a series of derivatives with optimized activity against SARS-CoV-2 and other coronaviruses.

## 6. Repurposing of Protease Inhibitors

**Figure 1:**
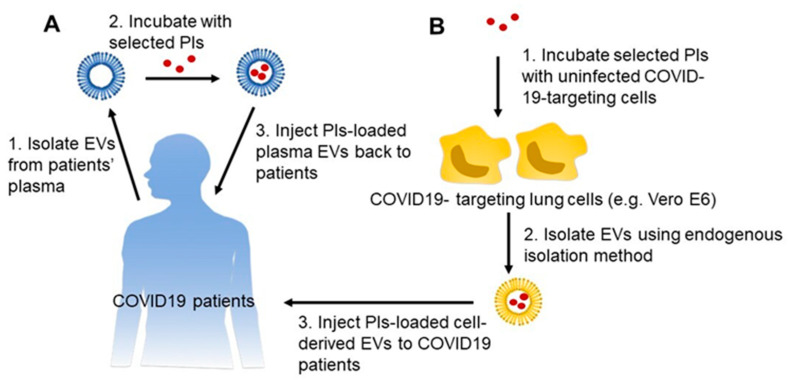
EV-based PI drug products as a treatment strategy to combat COVID-19. (**A**) Personalized medicine strategy: The EVs are isolated from the plasma of the patient, loaded with selected PIs, and administered back to the same patient using IV route. (**B**) Mass production strategy using endogenous loading: EVs are purified from cell culture media. PIs are incubated with uninfected cells/cell lines which target SARS-CoV-2. Isolate EVs with PIs already encapsulated for further treatment.

Anti-HIV drugs from the PI class could be more effective if targeted at the SARS-CoV-2-infected cells and/or at higher doses. However, a higher dose of PIs, if effective for COVID-19, would also cause toxicity and severe side effects, as most PIs are known to be toxic at higher doses [54]. In addition, being a substrate as well as an inhibitor of a major drug-metabolizing enzyme, cytochrome P450 3A4 (CYP3A4), these drugs cause drug–drug interactions (DDI) with approximately half of all marketed drugs [55,56]. Since COVID-19 has elevated symptoms and complications in elderly patients and in patients with underlying diseases such as heart problems, diabetes, and respiratory complications, PIs could cause major DDI, and ultimately, drug toxicity. Similarly, these drugs are substrates and inhibitors of a major drug efflux transporter, P-glycoprotein (Pgp), and also have the potential to cause DDI via the Pgp pathway [57]. Therefore, there is a need to bypass CYP3A4- and Pgp-mediated DDI and reduce toxicity to have an effective COVID-19 treatment, especially at high doses. Further, a unique drug delivery system, which has the ability to target the infected cells, would also provide increased drug concentrations at the target cells and reduced off-target effects (Figure 1).

To bypass drug efflux transporters and metabolic enzymes and target the disease site, a nano particle-based drug delivery system is highly desirable [57]. However, the use of chemical-based nanoparticles can be toxic, difficult to eliminate from the body, and may require FDA approval. On the other hand, extracellular vesicles (EVs) are membrane-derived nanovesicles that are circulated in extracellular body fluids, such as plasma and urine [58]. Therefore, for long-term safety and the ability to carry biological and therapeutic molecules to the target site, it is highly desirable to develop EVs as “biological nanoparticles” which are capable of delivering therapeutic agents to infected tissues [59]. The literature supports the development of an EV-based drug delivery platform for the potential use of therapeutic interventions in suppressing HIV reservoirs in the brain and reducing HIV-associated neurocognitive disorders (HAND) [60]. The literature also supports the application of an EV-based drug delivery system for the treatment of many cancers and CNS diseases/disorders [61].

## 7. Extracellular Vesicles as a Unique Drug Delivery System for Protease Inhibitors

Extracellular vesicles (EVs), produced from most cells/tissues/organs, are natural carriers for biological molecules, including DNA, RNA, miRNA, proteins, lipids, and other small molecules [62]. EVs are involved in many biological processes via intercellular communications, especially in the case of disease conditions, such as cancer, infectious diseases, and neurodegenerative disorders [63]. Being natural carriers of biomolecules, EVs may also be used as unique drug-delivery systems for drugs in a variety of diseases conditions [64]. EVs have already been used to encapsulate small molecules, nucleic acids, peptides, and proteins to treat different diseases, the majority of which are cancers [65]. Among anticancer agents, curcumin, doxorubicin, and paclitaxel are the top three drugs being investigated. For example, Kim et al. developed an EV-encapsulated paclitaxel to overcome multidrug resistance in cancer cells [66]. The EV encapsulation improved cytotoxicity more than 50-fold in drug-resistant cancer cells, compared with paclitaxel treatment alone. More importantly, an almost complete localization of EV-encapsulated paclitaxel was detected in a mouse model. EVs that can be used in drug delivery are mainly derived from the endolysosomal pathway, also called exosomes [67]. The common features of endolysosomal-derived vesicles/exosomes are that their size varies between 30 nm to 200 nm, and they contain endosomal-associated proteins, including tetraspanins CD9, CD63, and CD81 [68].

EV-based drug delivery provides an opportunity for utilization in personalized medicine, in which EVs can be isolated from the plasma of a patient, loaded with the drug(s) of interest, and administered back to the same patient [69] (Figure 1). This method is referred to as exogenous drug loading, and it can also be done in EVs isolated from the media of cells/cell lines [70]. This requires the isolation and characterizations of EVs of a particular size (<200 nm) from plasma or media, and it has the ability to interact with and deliver drugs to recipient cells [71]. Many drugs, especially nucleotides (mRNA, miRNA, siRNA, and dsDNA), have been loaded using exogenous techniques [72]. This technique utilizes direct incubation, sonication, electroporation, freeze-thaw, and extrusion methods [73]. A particular loading method is selected and optimized based on each drug of interest in terms of its size, shape, and needs for treatment outcomes.

To prepare a larger quantity of EV-loaded drugs for the general population and to target affected cells, one can load drugs in EVs using the endogenous drug-loading method. Endogenous loading is a method that uses cells to excrete EVs with target drugs encapsulated in them [74]. In endogenous loading, EVs already have target drugs loaded, once purified from cell culture media. This is done by incubating drugs with cells/cell lines that the disease targets, using the in vitro method. This method has the ability to scale up the technology for preparing a large quantity of drug-loaded EVs [64]. These drug-loaded EVs have the ability to target the diseased tissue, which is likely to have increased efficacy and reduced off-target effects compared with free drugs [75]. The endogenous drug-loading method has been used to load small molecules, especially anticancer agents [76]. This method has also been used to load nucleotides for various diseases [77,78,79].

There are at least eight PIs that have been developed and used alone or in combination with ritonavir for the treatment of HIV [80]. Some of these PI regimens are still being used as the first line of therapy in some parts of the world [81,82]. In some cases, these PIs, especially LPV/RTV and DRV/RTV, are used as the second line of therapy [83]. Thus, the development of an EV-based drug delivery platform for HIV PIs, individually and in combination with the pharmaco-enhancer RTV, is highly desirable. It is also possible that single protease inhibitor-loaded EVs are as effective as EVs loaded along with RTV. RTV strongly inhibits CYP3A4 and increases PI plasma concentrations. In principle, this would be unnecessary when the drugs are loaded in EVs, as EVs are likely to bypass CYP3A4. If successful, this drug delivery technology can be expanded to other existing antiviral and antiretroviral PIs or antibacterial drugs, which have shown some efficacy for treating COVID-19.

In the case of COVID-19, anti-HIV PIs, other PIs, or other antiviral and antibacterial drugs can be encapsulated in Vero CCL-81, Vero E6, and/or STAT1 knockout cell lines using the endogenous loading technique. These model cell lines have been developed by ATCC and can be infected with SARS-CoV-2 [84]. Drugs loaded in EVs and isolated from these cells would have the ability to target these same cells. PIs can also be loaded in EVs isolated from the plasma of patients, using the exogenous loading method for personalized therapy. The EV-encapsulated drugs can be first tested for efficacy in Vero CCL-81, Vero E6, and/or STAT1 knockout cell lines, followed by pharmacokinetics, tissue distribution, and finally efficacy, in an animal model. The lead formulations can then be used for human clinical trials at multiple sites. The clinical trials for repurposing PIs and other drugs using EVs could be very rapid because the drugs are already FDA-approved, and EVs are natural nanovesicles.

The literature suggests that EVs derived from a particular cell type, e.g., immune cells, can seek diseased and/or inflammatory immune cells by targeting the cell surface proteins [85]. Therefore, it is likely that EVs derived from SARS-CoV-2 model cell lines (e.g., Vero CCL-81 or Vero E6) have surface proteins that can recognize SARS-CoV-2-infected alveolar macrophages, and may secrete EV-encapsulated drugs in these cells. However, it is also possible that EV isolation and drug encapsulation techniques may result in the loss of the functional properties of the EVs, perhaps by destroying surface proteins. Thus, it is difficult to rule out nonspecific interactions of EVs with other cells, leading to off-target effects. If the off-target effects cause toxicity and suboptimal efficacy, EVs may need to be engineered to target SARS-CoV-2-infected cells. This can be done by engineering EVs, isolated from plasma, Vero CCL-81, or Vero E6 cells, with surface proteins or antibodies that can bind specific proteins on the target cells. In addition, new techniques may be developed to encapsulate these EVs in another biomimetic nanostructure, e.g., liposomes, for the delivery of EV-encapsulated drugs [86]. EVs have been shown to deliver their biological cargos via multiple mechanisms, such as the fusion of EVs with recipient cells and engulfing of EVs by a variety of recipient cells [87]. Although the mechanism of drug delivery to recipient cells by EVs has not been fully studied, it is likely that a similar mechanism to deliver drug-encapsulated EVs may also exist.

## 8. Production Feasibility and Compliance of Extracellular Vesicle-Based PIs

In most cases, EV-based drug products need to be administered in the form of injections. Hence, the sterility of the drug product is critical. EVs were recently shown to maintain stability under autoclave temperatures [88]. More importantly, EV-based drug products were reported to be eligible for sterile filtration using filters with 200 nm pore sizes [87]. Both autoclave and sterile filtration are FDA-approved methods for sterile drug preparation. Hence, EV-based PIs will be eligible for sterile drug manufacturing.

In the case of COVID-19, the FDA already granted orphan drug approval for remdesivir, even though Gilead rescinded. Additionally, the FDA is granting facilitated regulatory pathways for most clinical trial applications. Given their background, EV-based PIs might bypass the preclinical stage and move to Phase II directly. Because EVs are natural carriers with very solid safety data, Phase I studies might be waived by the FDA. The facilitated regulatory pathway is a strategy used by most regulatory agencies around the world to resolve pandemic diseases, including COVID-19, SARS, Ebola, etc. [89,90]. Such strategies will save time, resources, and in the best scenario, people’s lives.

## 9. Quality Control and Scalability of Production

EV-based drug products can be prepared through similar methods as lipid nanoparticles using exogenous loading methods. A typical life cycle of a potential EV-based drug product under cGMP standards should include production of EV raw materials, EV characterization, drug loading under cGMP conditions, in-process testing, fill-finish, final-release testing, and stability testing. Similar to lipid nanoparticle-based drug products, major quality control focuses on raw EV size distribution, postprocessing encapsulated drug amount, free drug amount, size distribution, zeta potential, and product stability.

The scalability of production is not a problem for current technologies. The bottleneck of EV-based drug production is drug loading into EVs. Current high-pressure homogenizers can process >100 L of drug liquid [91,92]. Furthermore, the ultracentrifugation technique can be used to process the elimination of >10 L of free drugs [93].

The University of Tennessee Health Science Center has an FDA-registered GMP facility named The Plough Center, with one coauthor working in it. This facility provides a top tier cleanroom environment and sterile injectable equipment, ensuring that EV-based drug production could be rapid, feasible, and may move forward to clinical stages [94].

## 10. Limitations of the Approach

Although EVs have the ability to circulate via plasma and deliver their components to distant tissues/organs, some general limitations are important to be addressed before realizing their clinical potential. These are: (1) interaction of drugs with EV components, (2) a robust understanding of how administered EVs are transported in vivo, (3) in vivo pharmacokinetics of EV-drugs, (4) delivery of drugs to the target, and (5) immune clearance [95,96]. Importantly, EVs have similar structures as liposomes, with two layers of lipids: a hydrophilic surface and core layer, with a hydrophobic membrane layer. Antiretroviral PIs are not water soluble, and therefore, these drugs will mainly reside inside the membrane due to the partition coefficient. However, unlike liposomes, we do not have a comprehensive understanding of how changes in size (30–150 nm), charge (zeta potential: −5 to −20), and the presence of specific surface proteins (based on the origin of EVs) affect their systemic circulation, overcoming of barriers (e.g., BBB), delivery of cargo to targeted tissues/organs, and clearance. Although EVs are expected to have poor entrapment in the plasma and clearance by the phagocytic system, which may lead to enhanced distribution and increased half-life, their biochemical complexity needs to be considered [75,97]. Furthermore, although strong preclinical evidence for systemic circulation and tissue/organ targeting exists for EVs (e.g., brain) [98,99], it is important to fully understand their transport and delivery mechanisms. However, in the case of COVID-19, we may not have sufficient time to perform comprehensive preclinical studies to understand the delivery of antiretroviral PIs to target tissues. Therefore, it is important that the FDA expedites the approval of an EV-based delivery method for PIs to treat COVID-19, upon determining its relative safety and efficacy compared with free drugs, in a small clinical trial.

## 11. Conclusions

COVID-19 is a global risk to humanity, and its impacts on health are yet to be determined. Since the development of new drugs and vaccines will take at least a year, it is critical that we consistently work on its mitigation until the world recovers from its spread. While the disease is spreading exponentially, especially in the US, several drugs are being tested to either reduce the symptoms of the disease or treat patients. Plasma treatment, by isolating antibodies from recovered patients and administering it to new patients in a controlled manner, is likely to be promising. Several drugs that were initially developed for other diseases have also been tried with patients, as well as tested in cell culture experiments, among which several antibody-based immunosuppressive drugs, the anti-CoV drug remdesivir, the antimalaria drug hydroxychloroquine, and the anti-HIV drugs lopinavir/ritonavir, have shown promising results. These drugs are therefore the subjects of further investigation in clinical trials for the treatment of COVID-19. There is also an initiative to repurpose anti-HIV PIs using unique drug delivery systems that utilize natural nanocarriers, EVs. These EV-encapsulated drugs have the ability to bypass liver metabolism and target diseased tissues, enhancing their efficacy and reducing off-target effects or drug toxicity.

## Figures and Tables

**Table 1:** Current drug candidates to treat COVID-19.

Classification	Drug Name	Dosage Form	Approved Indication	Potential Use in COVID19
Immunosuppressive drug	Tocilizumab	Injection	N/A	IL-6 receptor antagonist, reduce cytokine release syndrome- like features in severe patients.
Immunosuppressive drug	Sarilumab	Injection	N/A	IL-6 receptor antagonist, reduce cytokine release syndrome- like features in severe patients.
Immunosuppressive drug	Corticosteroids			reduce cytokine release syndrome- like features in severe patients.
Immunosuppressive drug	Baricitinib		For rheumatoid arthritis in adults who have had an inadequate response to one or more tumor necrosis factor (TNF) antagonist therapies.	A JAK inhibitor to reduce inflammation caused by cytokine storm.
Antiviral drugs	Hydroxychloroquine Sulfate	Oral (tablet)	Treatment of uncomplicated malaria	Block viral entry through the endo-lysosomal pathway.
	Lopinavir and ritonavir	Oral (tablet; solution)	HIV-1 protease inhibitor indicated in combination with other antiretroviral agents for the treatment of HIV-1 infection in adults and pediatric patients	Not clear
	Chloroquine Phosphate	Oral	Treatment of uncomplicated malaria	Block viral entry through the endo-lysosomal pathway.
	Remdesivir	Injection	Initially designed for protection against Ebola virus infection	Inhibit viral RNA synthesis
Antibiotic drug	Azithromycin (Injectable)	Injection	Community acquired pneumonia	Reduce pneumonia symptoms.
Blood Product, Antibody	Convalescent plasma	Injection	N/A	Use antibody-antigen strategy to eliminate virus.
